# Investigation of Ripple Formation on Surface of Silicon by Low-Energy Gallium Ion Bombardment

**DOI:** 10.3390/nano14131124

**Published:** 2024-06-29

**Authors:** Márk Windisch, Dániel Selmeczi, Ádám Vida, Zoltán Dankházi

**Affiliations:** 1 Department of Materials Physics, Eötvös Loránd University, 1117 Budapest, Hungary; mark.windisch@ttk.elte.hu; 2 Department of Development, Bay Zoltán Nonprofit Ltd. for Applied Research, 1116 Budapest, Hungary; adam.vida@bayzoltan.hu; 3 Semilab Semiconductor Physics Laboratory Co., Ltd., 1117 Budapest, Hungary; daniel.selmeczi@semilab.hu

**Keywords:** ion bombardment, silicon, ripple formation, amorphous layer, ellipsometry

## Abstract

Regular wave patterns were created by a 2 kV gallium ion on Si(111) monocrystals at incidence angles between 60° and 80° with respect to the surface normal. The characteristic wavelength and surface roughness of the structured surfaces were determined to be between 35–75 nm and 0.5–2.5 nm. The local slope distribution of the created periodic structures was also studied. These topography results were compared with the predictions of the Bradley–Harper model. The amorphised surface layers were investigated by a spectroscopic ellipsometer. According to the results, the amorphised thicknesses were changed in the range of 8 nm to 4 nm as a function of ion incidence angles. The reflectance of the structured surfaces was simulated using ellipsometric results and measured with a reflectometer. Based on the spectra, a controlled modification of reflectance within 45% and 50% can be achieved on Si(111) at 460 nm wavelength. According to the measured results, the characteristic sizes (periodicity and amplitude) and optical property of silicon can be fine-tuned by low-energy focused ion irradiation at the given interval of incidence angles.

## 1. Introduction

Parallel to the development of nanotechnology, ionic surface treatment became available which resulted in high-precision nanoscale machining of solids. One of these techniques is the focused ion beam (FIB) machining widely used in the micro- and nano-sized fabrication of materials. During ionic fabrication, such as thin lamella, micropillar preparation, undesirable micro- and nano-sized surface ripple formations can be observed. In our work, these unavailing periodic surface structures were first created, then tested by different microscopic and spectroscopic methods.

Firstly, Haymann and Trillat published ripple-like formation on electropolished uranium surfaces by low-energy argon ion bombardment in 1959 [1]. In addition to Haymann and Trillat, Cunningham and Navez observed grooves and ripple-like patterns on Au and the glass surface in the early 1960s [2,3]. Simultaneously with the first experimental results [4,5], theoretical works were also completed on this topic [6,7,8].

Due to major advances in ion beam machining in the 21st century, the number of experimental works increased significantly. Most experiments used noble gas ions such as Ne^+^, Ar^+^, Kr^+^, Xe^+^ [9,10,11,12,13]. These ion irradiations can also be grouped according to applied ion energy from a few tens of eV to a MeV-range energy treatment. According to published experimental works, ionic surface treatments were carried out mainly in the range of 100 eV to 100 keV ion energy. Periodic structures by ion bombardment were observed on some metals, semiconductors and insulators [14,15,16,17,18]. Due to the rapid development of the semiconductor industry, ion-irradiated Si, Ge and GaAs were also studied [19,20,21,22,23,24]. Several articles deal with surface ripples by noble gas ion irradiation [25,26,27,28,29]. The cited works examine the morphological properties (e.g., surface roughness, wavelength) of the created surface patterns as a function of the energy and incidence angle of the accelerated ions used. The results of studies on the subject demonstrate that certain properties of these modified surfaces can be controlled by the parameters used in ionic irradiation.

Along with some experimental works, the most cited phenomenological description is the Bradley–Harper (BH) model [30], providing an explanation of the formation of ripple structures. The BH model predicts the direction, wavelength and amplitude of formed ripples. The BH model predicted 90° ripple rotation of the structured surface at a given critical incidence angle. According to the model, the wave vector of the formed ripples is perpendicular to the direction of the incoming ion beam. Approaching the grazing incidence angle, below a critical one, the wave vector of the ripples becomes parallel to the incoming ions. This phenomenon has been experimentally observed on metals [31,32,33] and semiconductors at high temperature [34,35]. One of our goals is to test the BH model on a silicon surface at room temperature.

FIB is the “workhorse” ion irradiation technique used to shape micro- and nano-structures in materials processing. In previous works, 30 kV focused gallium ion beams created ripple formation on solids [36,37,38,39]. In these works, the periodicity of the structures is several hundred nanometers. It was observed that the wavelength of the waves created decreases as ion energy is reduced [27,40,41], so the sizes of the periodic structures are below 100 nm when these ripples are created by 2 kV ion beams. This can be also observed in experiments with several noble gas ions [42,43,44]. One of the aims of our experimental work is to investigate the periodic nanostructure created by a low-energy focused gallium ion beam, an arrangement missing from previous research. Furthermore, we took into account industrial developments and related applied research, such as the development of MEMS (micro-electro-mechanical systems) or other micro-components. Using a low-energy gallium ion beam, just a few square micrometers of nanostructured areas can be micromachined in a controlled manner. This primary advantage is in contrast to other ionic techniques (i.e., with Kaufman ion source) where size of the ion beam on the sample is in the range of a few mm to a few cm. Based on the size benefits, we are also looking for other optical properties that can be well controlled by ion irradiation and for their respective potential applications. One of the outcomes of our study was the controlled modification of the reflectivity of polished Si single crystal using a focused gallium ion beam.

## 2. Materials and Methods

The samples were commercially available polished Si(111) p-type substrates with a root-mean-square (rms) roughness of ∼0.2 nm. The samples were irradiated by 2 kV accelerated focused gallium ion beam using FEI Quanta 3D dual beam scanning electron microscope (SEM, Thermo Fisher Scientific, Hillsboro, OR, USA). During irradiation, the ion beam current and the ion dose were fixed at 4.3 nA (beam diameter ∼200 nm) and 1.2 · 10^17^ ion/cm^2^, respectively. The ionic surface treatment was performed at room temperature, at a pressure of 10^−4^ Pa in the chamber of the SEM. The morphology of the created structured surface was characterised by an AIST-NT Smart SPM-1000 atomic force microscope (AFM, Moscow region, Russia) operating in semicontact mode. The measurements were performed in air by µmash HQ:NSC19/Al BS AFM tip with a nominal tip radius less than 10 nm. The AFM measurements were performed with 2 μm× 2 μm scan size with a resolution of 512 × 512 pixels. The measured topography was analysed using the open source software Gwyddion (version 2.64) [45]. The characteristic wavelengths and roughness of the surfaces were calculated from plane-level and polynomial series-corrected measurements. The wavelength of ripple formations was determined using the position of the first peak of 1-dimensional power spectral density (PSD) functions which were calculated from the perpendicular direction square taken of 2-dimensional fast Fourier transformation (FFT) [46]. The local slope distribution was calculated from the in-plane *x* and *y* derivatives of the height values ($v=(\partial h_{x},\partial h_{y}$)). To represent the local slope distribution, the θ=arctan|v| was calculated. The sputtering yield was calculated using the SRIM2013 software [47]. The irradiation-induced crystal structure damage was determined with Semilab’s spectroscopic ellipsometer (FPT-u317). During the measurements, the effective spot size and the incidence angle of the light beam was 35 μm and 65°, respectively. The key to the analysis of the ellipsometric data is the selection of the correct optical model, fitting parameters and fitting algorithm. In order to have as few fitting parameters as possible (to avoid unwanted parameter correlations), the main layer was modelled using the Bruggeman effective medium approximation (EMA) widely used to describe rough surfaces [48]. Parameter fitting was performed using Semilab’s Spectroscopic Ellipsometry Analysis (SEA) software (version 1.8.0.4).

The reflection of amorphised surfaces was measured with Semilab’s spectroscopic reflectometer (FPT-mSR) with an effective spot size of 20 μm.

## 3. Results

**Figure 1:**
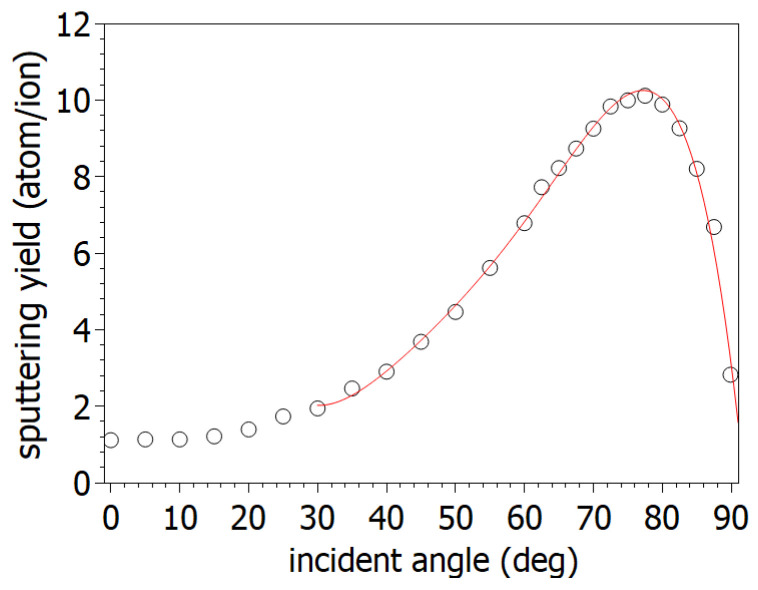
Simulated sputtering yield for the 2 kV Ga ion bombardment on Si by SRIM software [47].

Based on the SRIM simulation, the rate of increase in the sputtering yield on the Si surface during 2 kV Ga ion irradiation was the largest around 60–70° grazing angles (Figure 1).

**Figure 2:**
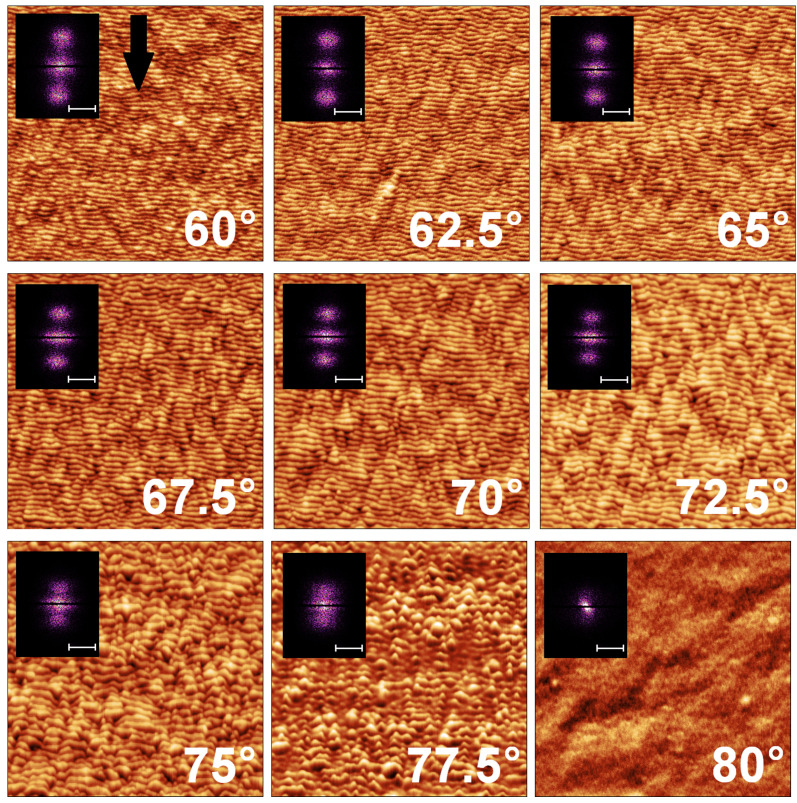
Morphology of 2 kV Ga ion irradiated 2 μm× 2 μm areas with their FFT images at different incidence angles with respect to the normal of the surface. (The direction of ion irradiation is indicated by a black arrow in the 60° AFM.) The scale bars in the FFT image represent 200 ${\mu m}^{-1}$. The height scale of AFM images: 60°: 4 nm, 62.5°: 6 nm, 65°: 7 nm, 67.5°: 9 nm, 70°: 11 nm, 72.5°: 14 nm, 75°: 17 nm, 77.5°: 13 nm, 80°: 3 nm. The ripple formation with increasing wavelength is parallel to the direction of the incidence ion beam in the range of 60° and 72.5°. The regular wave pattern breaks up between 72.5° and 77.5°, then a low roughness of smooth surface (rms roughness < 1 nm) forms.

Due to the ion incidence angle dependence of expected sputtering yield, the incidence angles of ion bombardment were changed from 60° to 80° with 2.5° resolution with respect to the normal vector of the surface. The topography of the irradiated surfaces is shown in AFM images (Figure 2). Based on the AFM measurements, ordered ripple formation evolved in the range of 60–75°. Above 75° irradiation, the regular ripple structures did not evolve but a slightly coarsened surface was formed.

**Figure 3:**
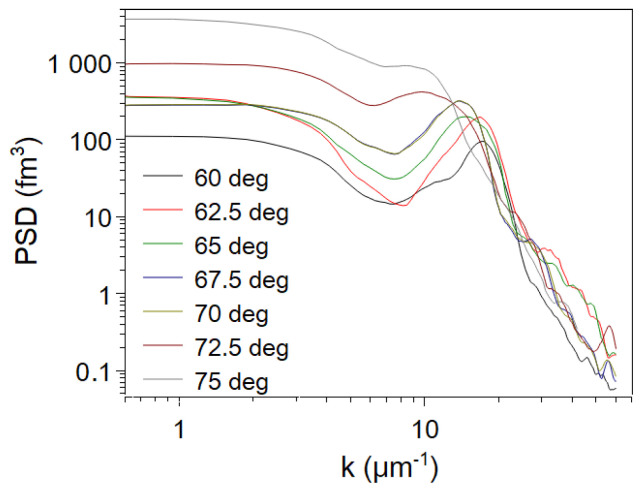
Power spectral density (PSD) functions of the evaluated AFM data of the irradiated surfaces. The first peak of the PSD functions shows the increasing wavelength of the periodic morphologies.

The wavelength of the ripple formation was calculated in the range of 60° to 75° using 2D FFT image of the AFM topography data. The PSD function was derived from the 2D FFT images providing sufficient accuracy for the periodicity of morphology. The increasing wavelengths of ripples can be observed in the offset of the first peak of PSD functions (Figure 3).

**Figure 4:**
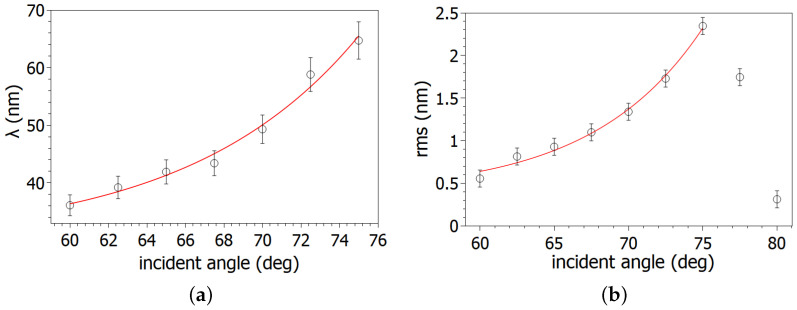
Quantitative analysis of the morphology of the irradiated surfaces: (**a**) wavelength (λ) and (**b**) rms roughness values (left and right side), respectively. The wavelength and rms values increase exponentially as a function of the ion incidence angles in the range of 60° to 75°.

The wavelength values and the rms roughness of morphology in the range of 60° to 75° show exponential growth in Figure 4.

The rms roughness and the simulated sputtering yield are maximum in the range of 75° to 77° ion incidence angle. The place of the maximum values are very close to each other indicating interdependence. For a more in-depth investigation of the surface roughness, the local slope distribution of the periodic surface was determined in the range of 60° to 80° ion incidence angles. The place of the maximum of the local slope distributions is shown in Table 1.

**Figure 5:**
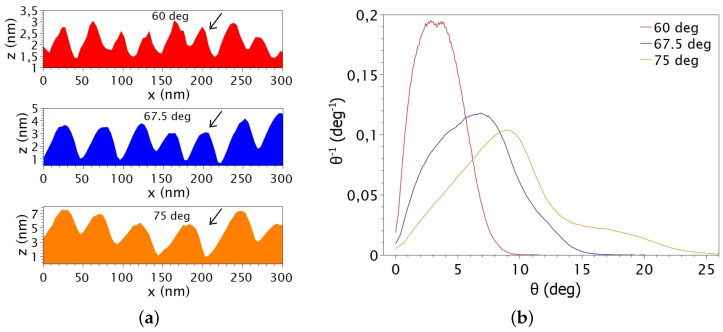
Representation of morphological analysis for irradiation at different angles: (**a**) cross section with direction of ion incidence beam (marked with black arrows) from AFM measurement of Figure 2 (left side) and (**b**) slope distribution (right side).

The maximum values of the local slope distribution are observed at an ion incidence angle of 75°. The trend is similar to the increase of roughness with increasing ion incidence angle. Based on these measured values and even taking into account the increasing wavelength values, the rms roughness can be represented by the amplitude of the respective wave patterns. The slope distribution of some structured surfaces at different ion incidence angles can be observed in Figure 5a.

It is important to note that as the ion incidence angles increases, an asymmetric peak appears around its maximum in the slope distribution (Figure 5b). This asymmetric peak is related to the different slopes of the wave pattern (Figure 5a). While the slope distribution at 60° has a nearly symmetrical peak, the cross section also shows the same curved wave crests. As the ion incidence angle increases, already at 67.5°, the wave crests show different slopes. It is known from the BH model that the sputtering depends significantly on the angle of curvature, which is the angle between the ion beam and the sample surface hit by incidence ions. Based on the BH model, the angle of curvature can well describe the initial inhomogeneities of the irradiated surface. The rate of this effect plays a role in the ripple formation process. Accordingly, there will be a different sputtering rate on a different side of the horizontal point (in this specific case, the angle of the incoming ion beam is the same as the actual local ion incidence angle) on the sample of the wave crest. As the local ion incidence angle, due to changes in the local curvature, approaches to the ion incidence angle at the maximum sputtering yield (Figure 1), the sputtering will increase. In other cases, as the local ion incidence angle decreases, the given part of the curvature is sputtered with less intensity. Based on the phenomenon described, there will be a “migration” of waves across the surface during the ion bombardment.

The 90° rotation of the ripple formation was not detected in the investigated 60–80° incidence angles. This observation is not consistent with the prediction of the BH model which predicts it, the given parameters of ion bombardment (mass of target and ion, accelerating of the ion), under 60° ion incidence angle. In other works, weak isotropic morphology was observed on silicon surface at near grazing incidence angles [26,49]. Additionally, the completed ripple rotation was detected only on chemically roughed silicon surface [50]. Though the BH model predicts the mentioned phenomenon, weak isotropic morphology appears on semiconductors, too. Our patterns did not display 90° ripple direction change on flat Si surface at room temperature.

**Figure 6:**
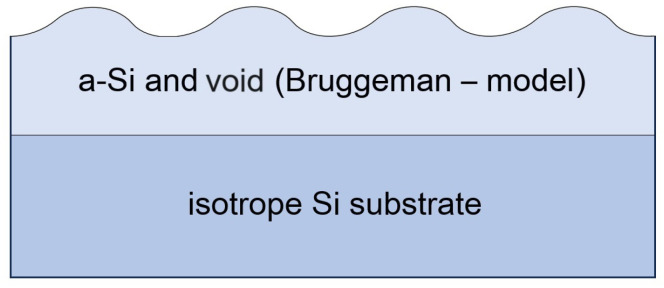
Applied model of the ellipsometric measurement of irradiated surface.

The amorphisation of irradiated surfaces was examined using spectroscopic ellipsometer (SE). The model stack consists of two layers. Considering the fact that in case of such thin layers, the unwanted correlation between fitting parameters can be strong, the native SiO_2_ layer does not present separately, since its thickness and optical properties are relatively close to the layer that we characterized. Instead, we used an effective model with only two fitting parameters which can reveal the changes between the optical properties of the samples. The amorphised thin layers were evaluated using Bruggeman’s effective approximation, which is quite a standard model for describing a roughness layer, using the mixture of void and a-Si dispersion (Figure 6). One can notice, the Tauc–Lorentz dispersion law can well describe amorphous silicon layer properties.

However, in this case, the parameters of the dispersion law are fitted during model creation and then kept fixed while analysing the different samples. Thus, comparing this dispersion law (still without the effect of void) to a-Si dispersion law might reveal information about the changes in 2–3 nm depth in 10 % gallium atoms (from SRIM simulations) contained on the top of the formed layer. The meaning of the dispersion law parameters could be considered as follows: *A* is the oscillator amplitude, $E_{0}$ is the oscillator peak position, *C* represents the width of the peak and $E_{g}$ is the band gap (Table 2).

**Figure 7:**
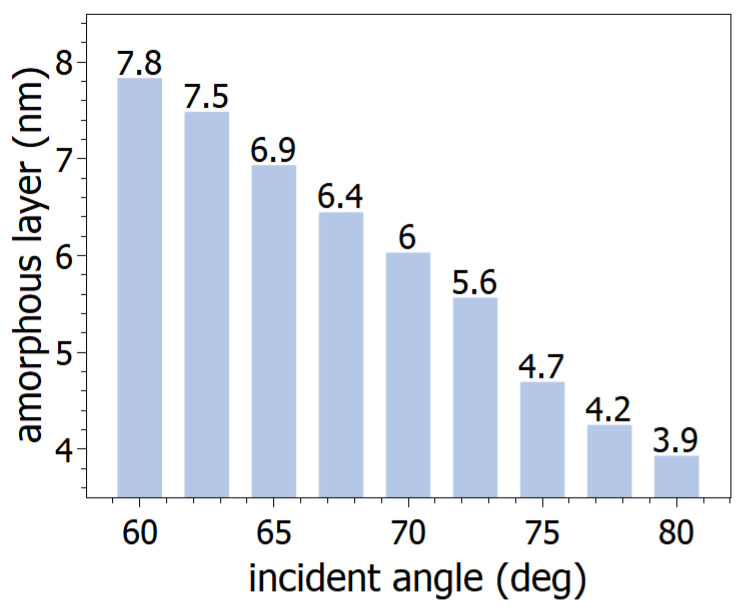
Thickness of the amorphous layer of the irradiated surface calculated from SE measurements.

The thickness and a-Si-void concentrations of the layer with the semi-infinite isotrope Si substrate were fitted in the analysis. Fitting of the model parameters (both in the case of model creation and fitting to all samples) is performed using the Levenberg–Marquardt algorithm by the SEA software. Based on the fitted results, the thickness of the damaged layers decreased from around 8 nm to 4 nm in the examined range of ion incidence angles (Figure 7).

**Figure 8:**
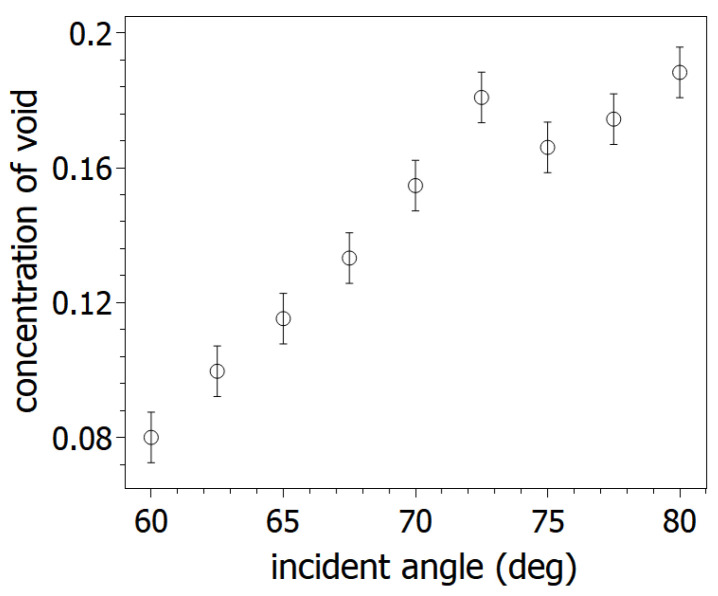
Proportion of void dispersion in the fitted SE models.

**Figure 9:**
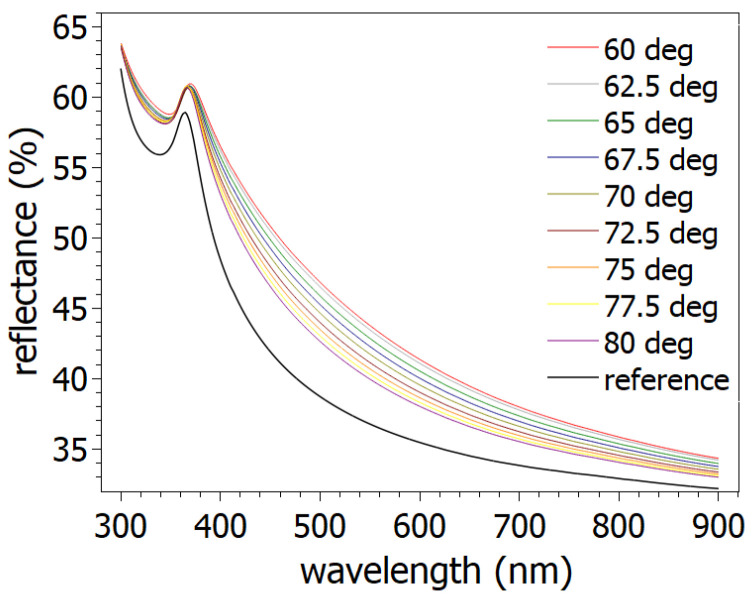
Simulated reflectances of irradiated surface in range of 60° to 80° and untreated polished silicon surface (reference).

Parallel to the thickness values, the void concentration of the layers shows growing tendency (Figure 8). The trend indicates that the effective density of the layer decreases versus the incidence angle. The *p* polarization reflectance of irradiated surfaces was simulated using fitted parameters of ellipsometric measurements (Figure 9). The simulated curves were checked by a reflectometer. The measured and simulated reflectance curves show good agreement. The correlation between measured and simulated values at 600 nm exceeds 93%. The measurement results gave the same trend as a function of the ion incidence angles as the simulated curves. The reflectance of the irradiated surfaces was examined in the range of 300 nm and 900 nm wavelength. The reflectance values decreased between 60° to 80° incidence angles. The decreasing tendency can be observed best at around 400 nm–600 nm light wavelength.

**Figure 10:**
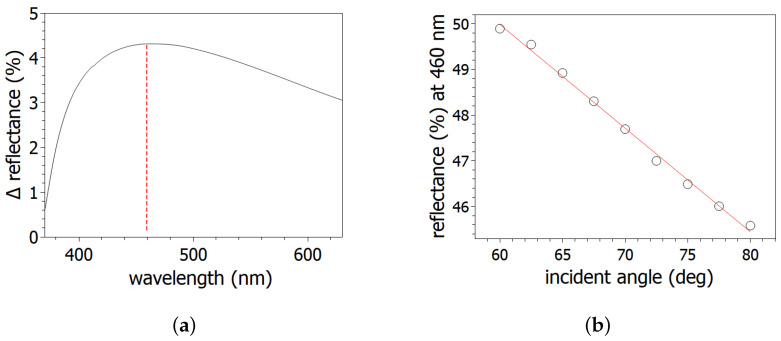
(**a**) Maximum reflectance difference (peak of the maxiumum marked red dashed line) of irradiated surfaces at 60° and 80°, and (**b**) reflectance values of irradiated surfaces at 460 nm applied light wavelength.

In order to determine the maximum difference of the modifications of reflectivity, the difference in reflectances (Δ reflectance) was plotted between 60° and 80° incidence angles in the wavelength range of 360 nm to 640 nm (Figure 10a). The maximum was observed around 460 nm. Based on that finding, reflectances at this 460 nm wavelength were plotted as a function of the ion incidence angles in Figure 10b.

These reflectance values show linear behaviour as a function of applied ion incidence angles. Due to its smooth monotonic dependence on the incidence angle, the reflectance of the Si(111) surface can be fine-tuned using 2 kV Ga ion irradiation.

## 4. Discussion

The characteristic sizes and shapes of the created wave pattern show similarities to other low-energy argon ion irradiation results [8,13,51,52]. Note that, in these cases, only part of the range of ion incidence angles takes the similarity into account. The asymmetric ripples of periodic patterns appear in low-energy work [13,49,53,54]. The local curvature dependence of the creation of ripple formation was examined as a function of the ion incidence angle. The evaluation shows that ripple formation is also influenced by the local curvature, consistent with the basic assumption of the BH model. Based on the model, the 90° ripple oreintation difference should have appeared under, respectively above, 60° ion incidence angle. However, in our experiments, the ripple’s “rotation” was not observed even around 80°. The reason for the lack of ripple rotation could be that during FIB machining, the surface is scanned by a few nanoampere, microwatt-range, 10–100 nm diameter ion beam; therefore, the increase in the temperature of the irradiated surface can be negligible during surface treatment. This is in contrast to other ionic surface treatments in which the beam diameter of a few millimeters is used in a continuous process. A continuous irradiation increase in the local temperature of the surface to an extent accelerates the surface diffusion process, leading to a change in the 90° ripple rotation. During the spectroscopic ellipsometric examination of the amorphisation of structured surfaces, a noticeable effect of a 2 kV gallium ion beam on the optical reflection was determined in the 60° and 80° ion incidence angles. Using the focused ion beam technique (with spot sizes of a few 10 or 100 nm), controlled modification of the optical properties of a few square micrometers of area has been achieved. This paves the way for new possibilities of nanotechnological surface modification in the semiconductor industry.

## 5. Conclusions

Ripple formation was observed on Si(111) due to low-energy focused gallium ion beam in the range of 60–80° ion incidence angles. The exponential behaviour of the wavelength and rms roughness values were determined by atomic force microscopy measurements. In addition, the local slope distribution and its maximum of the structured surfaces were determined. One of our future goals is to investigate the dependence of ripple formation on silicon crystallographic orientation by low-energy FIB. Based on the experimental works, the prediction of the BH model is not valid in our case. The amorphisation of the patterned surfaces was determined to be between 7.8 nm and 3.9 nm by spectroscopic ellipsometer. According to the results, increasing the void concentration of the damaged layer indicates that the effective density of the layer decreases as a function of the ion incidence angle. The reflectance of irradiated surfaces showed a decrease in the examined range of the ion incidence angles. Based on the results, close-to linear connection can be observed between the reflectance and ion incidence angle for 460 nm wavelength light. Due to the determined behaviour, a combination of the reflectance and the surface roughness of Si(111) can be fine-tuned (between 45% and 50%) by the 2 kV focused gallium ion beam. We aim to conduct further optical investigation of the irradiated areas and to explore potential industrial nano-applications of gallium ion FIB milling.

## Figures and Tables

**Table 1:** The location of the maximum of the local slope distribution (θ) as a function of the ion incidence angles.

Ion Incidence Angle (°)	θ (°)	$\theta_{\mathrm{error}}$ (°)
60	3.3	1.3
62.5	5.0	1.2
65	4.0	1.0
67.5	6.9	0.9
70	7.6	1.1
72.5	8.0	1.2
75	8.9	0.9
77.5	5.3	1.1
80	0.9	0.3

**Table 2:** Comparison of the Tauc–Lorentz parameters in the applied model and those used for the standard amorphous silicon.

Parameters	Amorphous Silicon	Tauc–Lorentz Model
*A*(eV)	220	83.69
$E_{0}$ (eV)	3.6	3.62
$C_{0}$ (eV)	2.7	2.68
$E_{g}$ (eV)	1.5	0.43

## Data Availability

Data are contained within the article.
